# Regulation of eosinophil functions by autophagy

**DOI:** 10.1007/s00281-021-00860-1

**Published:** 2021-05-21

**Authors:** Nina Germic, Aref Hosseini, Shida Yousefi, Alexander Karaulov, Hans-Uwe Simon

**Affiliations:** 1 Institute of Pharmacology, University of Bern, Inselspital, INO-F, 3010 Bern, Switzerland; 2 Department of Clinical Immunology and Allergology, Sechenov University, 119991 Moscow, Russia; 3 Laboratory of Molecular Immunology, Institute of Fundamental Medicine and Biology, Kazan Federal University, 420012 Kazan, Russia

**Keywords:** Autophagy, Differentiation, Degranulation, Eosinophil, Eosinophilic disease, Eosinophilic leukemia

## Abstract

Eosinophils are granule-containing leukocytes which develop in the bone marrow. For many years, eosinophils have been recognized as cytotoxic effector cells, but recent studies suggest that they perform additional immunomodulatory and homeostatic functions. Autophagy is a conserved intracellular process which preserves cellular homeostasis. Autophagy defects have been linked to the pathogenesis of many human disorders. Evidence for abnormal regulation of autophagy, including decreased or increased expression of autophagy-related (ATG) proteins, has been reported in several eosinophilic inflammatory disorders, such as Crohn’s disease, bronchial asthma, eosinophilic esophagitis, and chronic rhinosinusitis. Despite the increasing extent of research using preclinical models of immune cell-specific autophagy deficiency, the physiological relevance of autophagic pathway in eosinophils has remained unknown until recently. Owing to the increasing evidence that eosinophils play a role in keeping organismal homeostasis, the regulation of eosinophil functions is of considerable interest. Here, we discuss the most recent advances on the role of autophagy in eosinophils, placing particular emphasis on insights obtained in mouse models of infections and malignant diseases in which autophagy has genetically dismantled in the eosinophil lineage. These studies pointed to the possibility that autophagy-deficient eosinophils exaggerate inflammation. Therefore, the pharmacological modulation of the autophagic pathway in these cells could be used for therapeutic interventions.

## Introduction

Eosinophils are granulocytes which are characterized by their avidity for the acidic dye eosin [1]. Relatively few mature eosinophils are found in the peripheral blood of healthy humans (less than 400 per mm^3^) [2]. Moreover, under homeostatic conditions, eosinophils reside primarily in all regions of the digestive system except the esophagus [3]. Eosinophils are also present in lymphoid organs such as thymus, lymph nodes, and spleen. A physiological infiltration of eosinophils is also seen in non-lymphoid organs such as adipose and mammary gland tissues as well as in the uterus [4]. Moreover, the so-called regulatory eosinophils have been observed in normal human and mouse lungs [5]. In response to inflammatory stimuli, eosinophil differentiation in the bone marrow is increased and eosinophils migrate towards inflammatory tissues where their lifespan is prolonged [6, 7]. When blood eosinophil numbers exceed 400 per mm^3^, the term eosinophilia applies. A threshold of 1500 eosinophils per mm^3^ is usually employed to define blood hypereosinophilia [8]. A cytokine-independent eosinophilia is caused by genetic changes within the eosinophil lineage, and the resulting diseases are classified as primary or intrinsic eosinophilic disorders [9].

The most typical eosinophil characteristic which discriminates them from other granulocytes (neutrophils and basophils) is the presence of large specific granules in the cytoplasm [10, 11]. A significant amount of mediators, including cytotoxic cationic proteins, cytokines, chemokines, and growth factors, are preformed and stored within eosinophil specific granules, where they are available for a rapid, stimulus-dependent release [12, 13]. Other characteristic organelles in eosinophils are primary granules, known to contain the Charcot-Leyden crystal protein (also known as galectin-10), and lipid bodies, which are production sites of inflammatory lipid mediators such as cysteinyl leukotrienes, thromboxanes, and prostaglandins [14, 15].

Contrary to neutrophils, which are essential for host defense, pharmacological or genetic depletion of eosinophils does not cause evident functional consequences [16]. Nevertheless, the fact that eosinophil lineage in all vertebrates survived the evolution pressure over several thousands of years demonstrates the importance of eosinophils in health and disease [17]. While eosinophils have been traditionally perceived as cytotoxic effector cells, recent studies have revealed their additional immunomodulatory and homeostatic activities. The role of eosinophils in immunity is still a matter of dispute and remains to be ambiguously defined. Preclinical mouse models mimicking human eosinopenia [18, 19] and hypereosinophilia [20, 21] as well as the ability to specifically knockout genes in the eosinophil lineage [22] allow to investigate the potential roles of eosinophils in health and disease. Moreover, targeted anti-eosinophil therapies allow to draw conclusions regarding the contribution of eosinophils for physiological and pathophysiological processes in humans [23].

## Autophagy

**Fig. 1:**
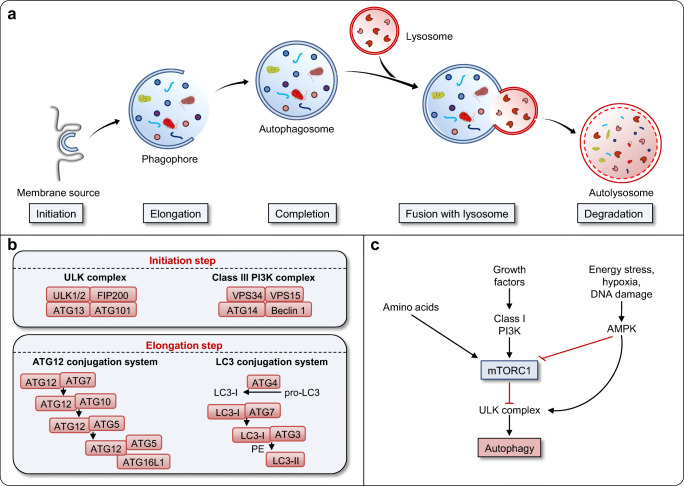
**Schematic representation of the mammalian autophagy pathway. a** Upon initiation of autophagy, a small portion of the cytoplasm is enclosed by the phagophore (also known as isolation membrane) which may originate from an ER-based structure. Elongation of the phagophore is followed by the autophagosome completion and its fusion with the lysosome, where the engulfed contents are degraded within the autolysosome. **b** ULK and class III PI3K (VPS34) protein complexes are required for the phagophore initiation. In addition, ATG12 and LC3 ubiquitin-like conjugation systems are necessary for subsequent elongation of the phagophore and its closure. **c** Autophagy is largely regulated by the activity of mTORC1, which reflects cellular nutritional status. Sufficient amounts of amino acids and growth factors suppress autophagy due to the inactivation of ULK complex by mTORC1 activity. On the contrary, mTORC1 activity is inhibited by cellular stress such as energy deprivation, DNA damage, and hypoxia, leading to the release and activation of the ULK complex which initiates the formation of autophagosomes

Macroautophagy (hereafter referred to as autophagy) is a highly dynamic intracellular degradation system by which cytoplasmic constituents are delivered to lysosomes for degradation [24, 25]. The main morphological feature of autophagy is the biogenesis of autophagosome, a unique double-membraned vesicle which encloses parts of the cytoplasm. The fusion between autophagosomes and lysosomes results in the formation of auto(phago)lysosomes, in which cargo is degraded by a number of hydrolytic enzymes. Breakdown products are then returned to the cytosol and reused [24, 25] (Fig. 1a).

The autophagic pathway is a central component of cellular stress response, and it assures a proper biological adaptation to the continually changing environment [26]. In general, autophagy has two principal functions: (1) provision of essential building blocks under the conditions of nutrient or energy deprivation and (2) maintenance of cellular homeostasis by elimination of damaged macromolecules and organelles [27]. Therefore, autophagy is a process which keeps individual cells in a healthy state. Any perturbation is expected to cause, or at least to contribute, to the pathogenesis of diseases. For instance, autophagy has been implicated in several pathologies, particularly neurodegeneration, cancer, inflammation, and infectious diseases [28–30]. Moreover, work in the last two decades has also demonstrated that autophagy is critical for multiple functions of the immune system such as removal of pathogens, differentiation of immune cells, antigen presentation, and regulation of inflammatory responses [31, 32].

The autophagic pathways have been characterized in excellent reviews, and general roles of autophagy in the regulation of immunity have been covered elsewhere [31–37]. Therefore, we only introduce the canonical autophagy pathway that requires autophagy-related (ATG) proteins. In the following, we discuss recently published work on the involvement of autophagy in eosinophil-associated diseases and review experimental work on the role of ATG proteins in eosinophil development and functions.

### The molecular mechanism of autophagy

Understanding of the molecular mechanism of autophagy has progressively advanced since the identification of *ATG* genes. In mammals, the protein machinery is organized into functional units which are required for autophagosome biogenesis. Upon autophagy induction, the ULK complex (consisting of the protein kinase ULK1/2, ATG13, ATG101 and FIP200) is generated, arising from regions where ATG9 vesicles line up with the endoplasmic reticulum (ER) [38, 39]. ULK complex is required for the recruitment and activation of the class III phosphatidylinositol 3-kinase (PI3K) complex (consisting of the lipid kinase VPS34, VPS15, ATG14, and Beclin 1), locally producing phosphatidylinositol 3-phosphate (PI3P) [39, 40]. The function of PI3P could be the formation of a phagophore membrane by modifying the composition of the ER membrane and recruitment of PI3P effectors [41]. Two ubiquitin-like conjugation systems are essential for proper elongation and closure of the phagophore membrane. The ATG12-ATG5-ATG16L1 complex is located mainly on the outer side of the phagophore membrane, followed by its disassembly upon autophagosome completion [41]. In the next step, the ATG12-ATG5-ATG16L1 complex promotes microtubule-associated protein light chain 3 (LC3) conjugation with phosphatidylethanolamine (PE) on the expanding phagophore membrane. Lipidated LC3 (LC3-II) serves as a commonly used marker for detection of double-membrane organelles owing to its integration into both the inner and outer autophagosomal membrane [42, 43] (Fig. 1b).

Autophagic pathway is strongly regulated by the activity of mechanistic target of rapamycin complex 1 (mTORC1), which senses the levels of nutrients and growth factors [44]. A strong trigger for autophagy induction is the lack of amino acids, leading to inhibition of mTORC1 activity and activation of suppressed ULK complex [45, 46]. Another central autophagy regulator is an AMP-activated protein kinase (AMPK), a sensor of metabolic, oxidative, and genomic stress [47]. Under the conditions of inadequate glucose levels, hypoxia or DNA damage, the AMPK activity is induced and promotes autophagy either by mTORC1 inhibition or direct phosphorylation of ULK complex [48, 49]. In addition, class I PI3K responds to growth factor signaling and suppresses autophagy by positively regulating the mTORC1 activity [50] (Fig. 1c). In contrast, class III PI3K (VPS34) forms a protein complex and produces the phospholipid PI3P, contributing to the initiation and progression of autophagy [50].

The protein p62 (sequestosome 1/SQSTM1) is a specific substrate for autophagy, and reduced autophagy is often related to excessive accumulation of p62 [41]. Besides, p62 selectively delivers ubiquitinated proteins to the phagophore membrane following the interaction with LC3-II [51, 52]. Finally, fusion between autophagosomes and lysosomes results in degradation of the auto(phago)lysosomal contents by hydrolytic enzymes [53]. Despite the extensive involvement of ATG proteins in autophagy machinery, it has been shown that many ATGs also exhibit additional non-autophagic functions that are discussed elsewhere [54, 55].

### Regulation of the function of immune cells by autophagy

The discovery of the ATG proteins was followed by in-depth investigation of the autophagy machinery and its critical involvement in the functioning of the immune system became evident [32]. The most direct approach of autophagy-dependent microbial removal is through xenophagy, a selective form of autophagy in which intracellular pathogens are targeted for autophagosomal sequestration [56–60]. Two possible mechanisms for the recognition of intracellular microbes by the autophagy machinery have been suggested. During xenophagy, the cytosolic bacteria are tagged by ubiquitin molecules and recognized by p62, which recruits the LC3-positive phagophores to capture the bacteria [61, 62]. Alternatively, ubiquitin is conjugated to host proteins on *Salmonella*-containing endosomes and binds with ATG16L1 independently of LC3-ubiquitin interaction through adaptor proteins [63]. In addition, p62 is able to deliver the ribosomal protein precursor Fau to autolysosomes where it is metabolized into bactericidal peptides. As a consequence, autophagic organelles are endowed with unique antimicrobial properties [64].

Autophagy is involved in the regulation of inflammatory responses. For example, it can suppress the immune response through the inhibition of inflammasomes, leading to reduced activation of caspase-1 and secretion of the pro-inflammatory cytokines IL-1β in IL-18 [65–69]. Moreover, autophagy has an influence on the homeostasis, survival, activation, proliferation, and differentiation of multiple cells of the immune system such as natural killer (NK) cells, macrophages, dendritic cells (DCs), as well as T and B cells. For instance, autophagy contributes to the maturation and antiviral activities of NK cells [70, 71]. Autophagy also participates in antigen presentation by DCs, forming a link between the innate and adaptive immune systems. The autophagic sequestration promotes the delivery and presentation of endogenous antigens on major histocompatibility complex (MHC) class II molecules, resulting in enhanced CD4^+^ T helper cell responses [72, 73]. On the contrary, autophagy enhances internalization and degradation of MHC class I molecules, leading to compromised MHC class I antigen presentation and attenuated response of cytotoxic CD8^+^ T cells [74]. Previous reports have also demonstrated the significance of autophagy for the development, survival and effector functions of T [75–79] and B cells [80–84]. Furthermore, it has been suggested that autophagy supports the release of β-hexosaminidase and histamine from mast cells [85]. The involvement of autophagy has also been demonstrated in neutrophil differentiation [86, 87] and effector functions [88].

In contrast to other cells of the immune system, the relevance of autophagy and ATG proteins for the biology of eosinophils has long remained elusive. However, insightful evidence was recently generated from experimental mouse models in which *Atg5* was specifically depleted in the eosinophil lineage, resulting in eosinophil-specific autophagy deficiency [89]. As discussed below, these models allowed testing the function of eosinophils under in vitro conditions in the presence and absence of ATG5. Moreover, these mouse lines were used in preclinical models of bacterial infection and primary hypereosinophilic disease.

## Role of autophagy in eosinophilic diseases

Interest in the role of autophagy in immunity was partially driven by the association of *ATG* genes with inflammatory disorders. Genetic alterations in autophagy may be hereditary, predisposing individuals to autoimmune, autoinflammatory, or infectious diseases. The involvement of autophagy has been observed in several eosinophilic inflammatory diseases, such as Crohn’s disease (CD), bronchial asthma, eosinophilic esophagitis (EoE), and chronic rhinosinusitis (CRS). Study models of eosinophilic inflammatory diseases and observed results are summarized in Table 1.

**Table 1:** **The involvement of autophagy and ATG proteins in eosinophilic inflammatory diseases.** Evidence for dysregulated autophagy has been reported in Crohn’s disease (CD), asthma, eosinophilic esophagitis (EoE) and chronic rhinosinusitis (CRS).

Disease	Study model	Observed results	Reference(s)
CD			
	CD patients with ATG16L1 SNP (rs2241880)	Increased susceptibility for CD (risk factor)	95, 96, 97, 98, 100
	CD patients with ATG16L1 SNPs (rs2241879, rs2241880)	Increased susceptibility for CD (risk factor)	99
	CD patients with ATG16L1 SNP (rs2241880)	Increased production of IL-1β and IL-6	101
	Atg16L1 Thr300Ala knock-in mice (rs2241880)	Enhanced caspase-3 - mediated degradation of Atg16L1, elevated IL-1β levels	102
	Atg16L1 Thr300Ala knock-in mice (rs2241880)	Defects in Paneth and goblet cells, elevated IL-1β levels, compromised host defense	103
	CD patients with IRGM SNPs (rs13361189, rs4958847)	Increased susceptibility for CD	100, 108, 110
	CD patients with IRGM SNPs (rs13361189, rs10065172, rs4958847)	Increased susceptibility for CD	106, 107
	CD patients with IRGM SNP (rs4958847)	Increased susceptibility for CD	109
	CD patients with ATG16L1 SNP (rs2241880) and IRGM SNP (rs10065172)	Genetic interaction contributes to CD pathogenesis	111
	Patients homozygous for ATG16L1 CD risk allele	Paneth cell granule abnormalities	112
	Atg16L1-deficient chimeric mice	Increased IL-1β and IL-18 levels, exacerbated inflammation in an experimental colitis model	65
Asthma			
	Asthma patients with ATG5 SNPs (rs510432, rs12201458)	Increased (rs510432) and decreased (rs12201458) susceptibility for childhood asthma	114
	Asthma patients with ATG5 SNP (rs12212740)	Increased susceptibility for asthma	116
	Severe asthma patients	Increased autophagy in blood eosinophils	117
	OVA-mouse model of severe asthma	Increased autophagy in BALF eosinophils	118
CRS			
	Atg7flox/floxLyzM-Cre mice	Increased eosinophil infiltration and inflammation	123
EoE			
	EoE patients	ATG7 as a novel tissue biomarker	125
	EoE mouse model	Increased autophagy with protective roles (decreased eosinophil infiltration)	126

### Crohn’s disease (CD)

Inflammatory bowel disease (IBD) encompasses two types of chronic inflammatory disorders of the gastrointestinal tract, CD, and ulcerative colitis (UC) [90]. Accumulating evidence suggests that inflammatory conditions in the intestine result from the abnormal immune response to enteric microbes in genetically predisposed individuals [91]. Genetic studies of IBD have made great progress since 242 risk loci have been identified through genome-wide association studies (GWAS) associated with the presence of IBD, highlighting some major disease-associated pathways [92]. Moreover, *NOD2* has been identified as a major susceptibility gene [93, 94].

Two genes involved in autophagy, *ATG16L1* and immunity-related GTPase M (*IRGM*), have been strongly associated with CD but not with UC, suggesting that autophagy is involved in the pathogenesis of CD [91]. A GWAS reported a single-nucleotide polymorphism (SNP) encoding a susceptibility variant of *ATG16L1* gene (rs2241880, Thr300Ala) which is associated with a significant risk for CD [95, 96]. Since its discovery, SNP rs2241880 remained one of the most clinically important variants in CD and a large number of subsequent association studies have replicated the strong association of this genetic variation with CD [97–100]. The *ATG16L1* Thr300Ala variant is associated with an excessive production of the pro-inflammatory cytokines IL-1β and IL-6 that drive the chronic inflammation observed in CD [101]. Moreover, this variant is more susceptible to cleavage by caspase-3, resulting in compromised clearance of *Yersinia (Y.) enterocolitica* and *Salmonella (S.) typhimurium*, as well as elevated cytokine production [102, 103].

IRGM has been demonstrated to induce autophagy and generate large autolysosomal organelles as a mechanism to inhibit the survival of intracellular *Mycobacterium (M.) tuberculosis* [104]. Recently, it has been reported that IRGM physically interacts with ULK1 and Beclin 1, promoting their assembly and thus controlling the arrangement of autophagy initiation complexes. In addition, IRGM forms a molecular complex with NOD2 and ATG16L1, modulating autophagic responses to pathogens [105]. A significant association has been reported between CD in various ethnic cohorts and sequence variants in the *IRGM* gene (SNPs rs13361189, rs4958847, and rs10065172) [100, 106–109]. The SNP rs13361189 was found to increase the risk of CD clinical sub-phenotypes such as ileal disease, perianal disease, and intestinal resection [110]. Interestingly, a gene-gene interaction analysis showed a significant two-way interaction between SNP rs2241880 (*ATG16L1*) and rs10065172 (*IRGM*), suggesting that ATG16L1 and IRGM work jointly toward CD pathogenesis [111].

Further insight into the role of ATG16L1 was obtained using genetically modified mice. Mice lacking *Atg16L1* in hematopoietic cells revealed a strong susceptibility to acute colitis induced by dextran sulfate sodium, implying that *Atg16L1* protects mice from intestinal inflammation [65]. *Atg16L1* is also important in the biology of epithelial Paneth cells as *Atg16L1*-knockout Paneth cells demonstrated a defective granule exocytosis which might alter the intestinal microbiota [112]. However, despite the intense investigations of the IBD pathogenesis, the role of autophagy in eosinophils has not been addressed yet.

### Bronchial asthma

Asthma is a heterogeneous disease characterized by chronic airway inflammation. Patients develop a variety of respiratory symptoms such as wheeze, breath shortness, chest tightness, cough, and expiratory airflow limitation [113]. Polymorphisms in *ATG* genes have suggested that a genetic predisposition may increase the chance to develop asthma. Since autophagy has been shown to regulate immune responses and inflammation, a possible association of genetic variants of *ATG5* and *ATG7* genes with childhood asthma was investigated. Two *ATG5* SNPs, rs12201458 and rs510432, were significantly associated with asthma, the latter being functionally relevant by enhancing promotor activity [114].

Moreover, *ATG5* gene expression was upregulated in nasal epithelial cells isolated from asthmatics with acute symptoms [114]. Similarly, the expression of ATG proteins (LC3-II, ATG4, ATG5-ATG12, ATG7) as well as the number of autophagic vacuoles was also increased in lung tissue from patients with chronic obstructive pulmonary disease (COPD) [115]. Another study investigated the potential association of SNPs in *ATG* genes (*ULK1, SQSTM1, MAP1LC3B, BECN1*, *and ATG5)* with asthma. SNP rs12212740 of *ATG5* exhibited a positive association with asthma [116]. Examination of bronchial tissue from asthmatic patients demonstrated an increased number of autophagosomes in fibroblasts and epithelial cells compared with healthy individuals [116].

Similarly to structural cells, autophagy levels in sputum granulocytes, blood leukocytes, and blood eosinophils from patients with severe asthma were significantly increased as compared with subjects with non-severe asthma and healthy controls [117]. Interestingly, autophagy was induced in isolated blood eosinophils and human eosinophil-like (HL-60) cells in response to IL-5 treatment. To confirm that IL-5 induced autophagy rather than inhibited autophagosome degradation, inhibitors were used which blocked autolysosome degradation or fusion of autophagosome with lysosome [117]. These findings stimulated investigations in ovalbumin (OVA)-specific mouse model of allergic asthma [118]. OVA-challenged mice exhibited an increased expression of LC3-II in lung homogenates and a higher abundance of autophagosomes in cells of the bronchoalveolar lavage fluid (BALF), particularly in eosinophils. The eosinophil count in BALF also positively correlated with the LC3-II expression in lung homogenates, suggesting that autophagy is closely correlated with the severity of asthma as well as the eosinophilic inflammation. Inhibition of autophagy by intraperitoneal injection of 3-methyladenine (3-MA) and intranasal treatment with *Atg5* shRNA led to a significantly improved airway hyperresponsiveness (AHR), reduced number of eosinophils and IL-5 levels in BALF, as well as improved histological inflammatory features [118]. However, it cannot be excluded that 3-MA also blocked cytokine signaling events in this model [119, 120]. Finally, intranasal administration of anti-IL-5 monoclonal antibody resulted in reduced LC3-II expression in lung homogenates, together with improved AHR and decreased eosinophil numbers in BALF [118]. Taken together, there is evidence that autophagy is induced in structural and inflammatory cells of the lungs in asthma, but it remains unclear how this phenomenon contributes to the pathogenesis of asthma. Therefore, it seems too early to propose novel therapeutic approaches for the treatment of asthma based on autophagy inhibition.

### Chronic rhinosinusitis (CRS)

CRS is characterized by chronic inflammation of the sinonasal mucosa and clinically associated with sinus pressure, nasal congestion, and a decreased sense of smell persisting for more than 12 weeks [121]. CRS is often associated with pronounced eosinophil-dominant infiltration and inflammation and then classified as eosinophilic chronic rhinosinusitis (ECRS) [122].

The effect of autophagy on the development of ECRS was investigated in mice with a conditional knockout of *Atg7* within myeloid cells (mainly neutrophils and macrophages), which was mediated using the LyzM-*Cre* (Lyz2-*Cre*) recombinase activity. An established mouse model of ECRS resulted in significantly increased eosinophil infiltration, epithelial hyperplasia, and mucosal thickening in *Atg7*^flox/flox^LyzM-*Cre* mice as compared with *Atg7*^flox/flox^ mice, possibly owing to increased prostaglandin (PG) D_2_ production [123]. Interestingly, eosinophil infiltration and histological abnormalities were significantly improved following macrophage depletion in *Atg7*^flox/flox^LyzM-*Cre* mice with ECRS. The autophagy-deficient macrophages exacerbate the eosinophilic inflammation in ECRS, at least partially, through the release of elevated IL-1β levels. Therefore, results of this study suggest a protective role of macrophage autophagy on eosinophilic inflammation [123].

### Eosinophilic esophagitis (EoE)

EoE is defined as a chronic, immune-mediated disorder resulting in esophageal dysfunction and eosinophil-predominant inflammation leading to tissue remodeling and fibrotic stricture [124]. A study performed on a pediatric patient cohort revealed upregulated *ATG7* gene expression in esophageal biopsies from active EoE patients as compared with esophagus-healthy control individuals, EoE patients in remission and patients with gastroesophageal reflux disease (GERD) [125]. Therefore, ATG7 might be used as a valuable tissue biomarker of active EoE, and other *ATG* genes may be explored to potentially identify novel biomarkers for EoE diagnosis, monitoring, and prognosis. A recent study revealed a possible cytoprotective mechanism of autophagy which supports cellular redox balance and homeostasis following exposure to the inflammatory EoE environment, providing mechanistic insights into the role of autophagy in EoE pathogenesis [126]. Specifically, TNF-α and IL-13 have been identified as triggers of autophagy within the epithelium of the esophagus under in vitro conditions, including an esophageal organoid model. Inhibition of autophagic flux *via* chloroquine treatment augmented basal cell hyperplasia in these model systems. Moreover, this study has demonstrated increased autophagy in epithelial cells of the esophagus in EoE patients in vivo [126]. However, it should be noted that also this study did not investigated the role of autophagy in eosinophils.

## Role of autophagy in eosinophil differentiation

Understanding the differentiation of eosinophils is crucial since many eosinophilic diseases are associated with increased production of eosinophils in the bone marrow. Eosinophils are continuously produced from eosinophil lineage-committed progenitors (EoPs) which derive from common myeloid progenitors (CMPs) in human [127] and granulocyte-monocyte progenitors (GMPs) in mice [128]. Terminally differentiated eosinophils are no longer mitotically active, and they are released into the circulation [129]. To keep cellular homeostasis, it is important that the differentiation process of eosinophils is tightly controlled [130].

Eosinophil differentiation is regulated by a complex network of transcription factors and extrinsic signals. During eosinopoiesis, a unique set of dynamic changes in the expression of transcription factors occurs, among which GATA-1, PU.1, and C/EBP members are the most critical ones [131, 132]. In addition, RhoH (small atypical GTPase) has been reported as a negative regulator of eosinophil differentiation, presumably due to dysregulated GATA-2 expression [133]. XBP1 has been identified as a highly selective and required transcription factor for eosinophil development [134]. It supports the survival of cells of the eosinophil lineage in a cell-intrinsic way while having no effect on basophils or neutrophils [134]. IL-5, IL-3, and granulocyte/macrophage colony-stimulating factor (GM-CSF) are recognized as the most relevant cytokines which stimulate eosinophil differentiation, activation, and survival [135]. A recent study implies IL-33 in the control of various stages of eosinophil differentiation mainly through the expansion of eosinophil precursors and upregulation of the IL-5 receptor α (IL-5Rα) on this population [136].

Until recently very little was known about the role of autophagy in eosinophils. Two studies investigated the involvement of mechanistic target of rapamycin (mTOR) on eosinophil hematopoiesis and asthma pathogenesis [137, 138]. The mTOR functions as a major nutrient-sensitive regulator of cell metabolism, balancing many anabolic and catabolic processes including protein synthesis and autophagy, respectively [44]. mTOR is a serine/threonine protein kinase that forms two distinct protein complexes, mTORC1 and mTORC2 [44]. Rapamycin selectively inhibits the activity of mTORC1, resulting in restricted eosinophil differentiation from mouse bone marrow cells and reduced cytokine production in vitro [137]. To examine the effect of rapamycin on allergic asthma, rapamycin was administered in a mouse model of OVA-induced allergic airway inflammation. Eosinophil numbers in BALF, peripheral blood, and bone marrow of rapamycin-treated mice were remarkably decreased, resulting in attenuated allergic airway inflammation and mucus production. However, rapamycin induced the accumulation of eosinophil progenitors in the bone marrow [137].

The same group subsequently investigated the function of mTOR in eosinophil differentiation and asthma pathogenesis using both genetic and pharmacological approaches. Treatment with torin-1, an inhibitor of mTORC1 and mTORC2, resulted in an enhanced in vitro eosinophil differentiation, as well as an increased size and number of eosinophil colony-forming units [138]. Similarly, *Mtor*^flox/flox^LyzM-*Cre* mice with a myeloid-specific knockout of mTOR exhibited an augmented production of eosinophil progenitors together with deteriorated allergic airway inflammation after OVA exposure [138]. Collectively, these data demonstrated the differential effects of mTOR in the regulation of eosinophil development, likely due to the distinct functions of mTORC1 and mTORC2 [138].

**Fig. 2.**
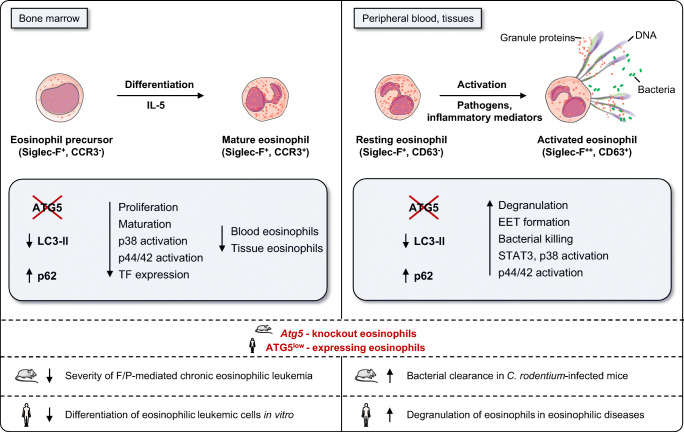
**The role of ATG5 in eosinophil differentiation and effector functions.** *Atg5*-knockout eosinophil precursors with suppressed autophagy exhibit a delayed and reduced proliferation, maturation, p38 and p44/42 MAPK activation, and a reduced expression of *Gata-1*, *C/ebpε*, *Pu.1,* and *Trib1* transcription factors (TFs). A decrease in eosinophil differentiation results in reduced numbers of mature eosinophils in blood and peripheral tissues. The differentiation capacity of eosinophil precursors in the absence of *Atg5/ATG5* is reduced in established mouse (FIP1L1-PDGFRα; *F/P* mice) and human eosinophil leukemia models (EoL-1 cells). Moreover, *Atg5*-knockout eosinophils exhibit enhanced degranulation, EET formation, bacterial killing, and signaling transduction following their activation in vitro*.* Mice with *Atg5*-knockout eosinophils have been shown to better clear a bacterial infection with *C. rodentium*. ATG5^low^-expressing human eosinophils demonstrate enhanced degranulation abilities in both tissues and blood

With the purpose of studying the developmental and functional consequences of autophagy deficiency in eosinophils, a novel mouse model with an eosinophil-specific knockout of *Atg5* was generated (*Atg5*^flox/flox^eo*Cre* mice) [120]. *Cre* recombinase is expressed only after commitment to the eosinophil lineage and is absolutely specific for eosinophils (eo*Cre* mice) [22]. Results obtained from *Atg5*^flox/flox^eo*Cre* mice showed elevated numbers of immature eosinophils in the bone marrow and a significant drop of mature eosinophils in the circulation [89] (Fig. 2). Knockout of *Atg5* within eosinophils resulted in delayed and reduced eosinophil precursor proliferation and maturation under in vitro conditions. No abnormalities in cell death were observed in *Atg5*-knockout eosinophils. During in vitro eosinophil differentiation, a reduced phosphorylation of p38 and p44/42 mitogen-activated protein kinases (MAPKs) was detected in eosinophil precursors lacking *Atg5,* which might explain, at least partially, the observed phenotype. Eosinophil populations were further purified from the bone marrow of hypereosinophilic *II5* (IL-5) transgenic mice (NJ.1638), showing downregulation of *Gata-1, C/ebpε*, *Pu.1*, and *Trib1* transcription factors in the absence of *Atg5*, which might reflect the reduced and delayed eosinophil differentiation [89]. Moreover, the differentiation potential of *Atg5*-knockout eosinophil precursors was tested under pathologic conditions, employing an established mouse model of chronic eosinophilic leukemia (CEL). CEL has been initiated in mice by the combination of fusion protein FIP1L1-PDGFRα (F/P) expression and IL-5 overexpression [139], resulting in a less severe eosinophilia development in the absence of *Atg5*. Similar results were obtained in EoL-1 cells, a model of an established human CEL. Upon induced differentiation of EoL-1 cells, significantly lower levels of surface markers CD11b, Siglec-8 and CCR3 were observed in *ATG5*-knockout EoL-1 cells, indicating decreased maturation of eosinophil precursors [89] (Fig. 2). These observations suggest that targeting ATG5 within the eosinophil lineage might represent a possible future treatment of eosinophilic leukemia.

These findings were in contrast with the effect of *Atg5*-deficiency on neutrophil differentiation in *Atg5*^flox/flox^LyzM*-Cre* mice [86]. These mice exhibited an increased proliferation and differentiation of *Atg5*-knockout neutrophils, culminating in neutrophil accumulation in the circulation and lymphoid organs. Accelerated neutrophil differentiation was also observed upon shRNA-mediated *Atg5* knockdown in Hoxb8 neutrophils [86]. Furthermore, autophagy was studied in early granulopoiesis in mice with a conditional deletion of *Atg7* at the hematopoietic stem and progenitor level (*Atg7*^flox/flox^Vav-*Cre*), which caused accumulation of immature neutrophils in the spleen, blood, and peritoneum [87]. An expanded population of immature myeloblasts and myelocyte precursors was also observed in *Atg7*^flox/flox^Cebpa-*Cre* mice, expressing *C/ebpα* promotor predominantly at the GMP stage. *Atg7*-knockout neutrophil precursors were unable to shift from glycolytic activity toward mitochondrial respiration, demonstrating accumulation of lipid bodies and decreased ATP production. Inhibition of autophagy-mediated lipid degradation failed to provide free fatty acids to support mitochondrial respiration and ATP production, resulting in a defective neutrophil differentiation [87].

Interestingly, it has been reported that the p38 MAPK activity differentially regulates eosinophil and neutrophil differentiation, potentially through the modulation of C/EBPα transcriptional activity [140]. A recent study showed that Trib1 expression favors eosinophil development by restraining neutrophil lineage commitment by modulating C/EBPα, partly clarifying the regulation of granulocytic lineage selection and identity [141]. In addition, single-cell transcriptome analysis has determined two different myeloid progenitor subsets which separate early in the hematopoietic development. Subsets can be differentiated according to the presence (eosinophils, mast cells, megakaryocytes, erythrocytes) or absence (neutrophils, lymphocytes, monocytes) of *Gata1* expression [142]. The potential involvement of autophagy in the segregation and regulation of these two distinct myeloid progenitor-differentiation pathways has not been established yet and could be the subject for future studies.

## Role of autophagy in eosinophil effector functions

Activated eosinophils exert their effector functions mainly through degranulation and formation of eosinophil extracellular traps (EETs). While both degranulation and EETs are important innate immune effector functions against pathogens, they can also cause significant immunopathologies [14].

### Degranulation

Eosinophil specific granules are rich in four major cationic proteins: major basic protein (MBP), eosinophil peroxidase (EPX), and the ribonucleases eosinophil cationic protein (ECP) and eosinophil-derived neurotoxin (EDN) [143]. Eosinophils release granule contents into the extracellular space through three commonly observed pathways, namely, piecemeal degranulation (PMD), exocytosis, or cytolysis [17]. During PMD, the specific granules are progressively emptied and eosinophil sombrero vesicles (EoSVs) transfer the selected secretory cargo to plasma membrane [144, 145]. Granules can release their entire contents following granule fusion with the plasma membrane in a process called exocytosis, which can be preceded by intracellular granule-granule fusion [146]. Eosinophil cytolysis is characterized by the disintegration of cytoplasmic membrane and release of nuclear DNA cloud together with intact granules, which are deposited in tissues upon cell lysis [147]. It has been demonstrated that eosinophil cytolysis is dependent on receptor-interacting protein kinase 3 (RIPK3)-mixed lineage kinase-like (MLKL) signaling pathway and can be counterregulated by autophagy induction, perhaps opening up new ways for therapeutic interventions [148]. PMD and cytolysis have been frequently reported to be associated with eosinophilic diseases such as asthma, nasal polyps, IBD, allergic rhinitis, and EoE [149–151]. On the contrary, exocytosis is rarely documented during inflammatory responses, but has been observed during the interaction of eosinophils with helminths [152] and certain environmental fungi [153].

The effect of autophagy on eosinophil degranulation has been studied in *Atg5*-knockout eosinophils, which intriguingly exhibited an enhanced capacity to degranulate in vitro as measured by increased CD63 surface expression following GM-CSF priming and C5a stimulation [89] (Fig. 2). Moreover, an experimental in vivo mouse model of bacterial infection with *Citrobacter (C.) rodentium* was used to test the infiltrating colonic eosinophils for their activation and degranulation status. Eosinophils lacking *Atg5* exhibited higher Siglec-F and CD63 surface expression levels, supporting the in vitro observations [89]. In addition, the expression of ATG5 in human eosinophils was analyzed together with their degranulation status in human eosinophilic tissues. Patients with angiolymphoid hyperplasia, EoE, and sebaceous gland carcinoma demonstrated a positive correlation between ATG5 expression and intracellular EPX levels in tissue eosinophils, suggesting increased degranulation in ATG5^low^-expressing eosinophils. These data were supported by observations in hypereosinophilic syndrome (HES) patients, in which a significant negative correlation between *ATG5* (and *ATG7*) mRNA expression in blood eosinophils and secreted EDN levels in plasma was found [89] (Fig. 2). These data supported the concept that *Atg5*-knockout eosinophils in mice and ATG5^low^-expressing human eosinophils are more susceptible to degranulation.

In contrast to eosinophils, autophagy was reported to be crucial for the degranulation of mouse neutrophils [88] and mast cells [85]. Mice with autophagy deficiency in myeloid cell lineage (*Atg7*^flox/flox^LyzM-*Cre*) showed reduced severity of several neutrophil-mediated inflammatory and autoimmune disease models, including PMA-induced ear inflammation, LPS-induced breakdown of blood-brain barrier, and experimental autoimmune encephalomyelitis. The most likely mechanism was suggested to be a reduced NADPH oxidase-mediated production of reactive oxygen species (ROS) in *Atg7*-knockout neutrophils [88]. These findings suggest that autophagy has differential effects on eosinophil and neutrophil degranulation. It has also been reported that in contrast to neutrophils, which are absolutely required for antibacterial defense, pharmacological or genetic ablation of eosinophils does not result in obvious functional consequences [16].

### Extracellular trap (ET) formation

Eosinophils are able to form ETs which consist of mitochondrial DNA and cationic granule proteins released from activated cells [154]. EETs perform antibacterial functions, and they enable the accumulation of toxic granule proteins directly onto pathogens captured in the DNA scaffold, limiting the damage of surrounding host tissues [155]. The formation of EETs has been demonstrated in various infectious, allergic, and autoimmune eosinophilic disorders [154, 156–159].

In addition to eosinophils, activated neutrophils are also able to form similar extracellular DNA structures, known as neutrophil extracellular traps (NETs) [160, 161]. Interestingly, the release of mitochondrial DNA does not require cell death neither does it limit the viability of the granulocytes [161]. On the other hand, neutrophil death-dependent mechanisms have also been described, and the scientific dispute regarding the requirement of cell death for NET formation is ongoing [155]. It has been demonstrated that NET formation by viable neutrophils depends on the activity of NADPH oxidase, cytoskeleton rearrangements, and glycolytic ATP production [162, 163].

A recent study reported the requirement for autophagy in the formation of EETs in the airway of asthmatic mice. Treatment with the autophagy inhibitor 3-MA attenuated EET formation and improved the lung inflammation, mitochondrial metabolism, and oxidative stress in OVA-challenged mice [164]. Moreover, treatment of neutrophils with autophagy inhibitor wortmannin reduced NET formation by activated neutrophils [165, 166]. 3-MA and wortmannin have been widely used as autophagy inhibitors based on their inhibitory effect on class III PI3K activity, which is known to be essential for autophagy induction [50]. The same inhibitors, however, are also reported to block class I PI3Ks which contribute to the activation of NADPH oxidase [167–169]. Therefore, decreased formation of EETs following 3-MA and wortmannin treatment can also be explained by inhibition of ROS production. A recent study has investigated this issue in details, and the reported results suggest that PI3K inhibitors, such as 3-MA and wortmannin, block both EET and NET formation in an autophagy-independent manner [120]. In addition, both *Atg5*-knockout eosinophils and *Atg5*-knockout neutrophils were fully capable to release extracellular DNA after stimulation, demonstrating that autophagy is not required for both EET and NET formation [120]. A careful quantitative analysis revealed that *Atg5*-knockout eosinophils exhibit an even increased ability of EET formation compared with control eosinophils in vitro, supporting the surprising findings of augmented degranulation in eosinophils lacking *Atg5* [89]. Eosinophils lacking *Atg5* also demonstrated elevated in vitro bacterial killing of *Escherichia (E.) coli*, suggesting increased effector function of eosinophils in the absence of *Atg5*. Moreover, the antibacterial defense of *Atg5*-knockout eosinophils was tested under in vivo conditions in the *C. rodentium* model. An improved local and systemic clearance of *C. rodentium* was observed in *Atg5*^flox/flox^eo*Cre* mice, together with an enhanced ability to form EETs [89]. *Atg5*-knockout eosinophils demonstrated an increased activity of Stat3, p38, and p44/42 signaling pathways following cytokine stimulation, providing a possible explanation for enhanced eosinophil effector functions in the absence of *Atg5* [89].

Tumor-associated tissue eosinophilia is often observed in cancer patients and studies suggest their involvement in tumoricidal activities. Upon interaction with a colorectal carcinoma cell line Colo-205, eosinophils released their granule contents such as ECP, EDN, TNF-α, and granzyme A, which exerted cytotoxic responses against tumor cells [170]. Moreover, ablation of eosinophils severely compromised antitumor immunity in a colorectal cancer (CRC) mouse model, most likely owing to impaired Th1 and CD8^+^ T cell responses. On the other hand, CRC patients with enhanced eosinophil tumor infiltration demonstrated robust CD8 T cell infiltrates, resulting in a better prognosis compared with patients with low-eosinophil infiltrating tumors [171]. Interestingly, the blockade of autophagy enhanced T helper 9 (Th9) cell anticancer functions in vivo, and mice with T cell-specific deletion of *Atg5* exhibited reduced tumor growth in an IL-9-dependent manner [172]. Finally, it would be interesting to investigate the functional consequences of *Atg5*-knockout eosinophils in different disease models and explore their potential for cancer immunotherapy.

## Concluding remarks

The findings summarized in this review article highlight the autophagic pathway as a protective mechanism of cells which contributes to the limitation of disease severity in eosinophilic diseases. Autophagy seems to secure the function of parenchymal cells under inflammatory conditions. This process appears to be particularly important in epithelial cells to maintain their barrier function. The generation of conditional/promoter-specific knockout mice has enabled researcher to investigate the role of autophagy in a cell-type specific manner. Recently, such experimental models were also developed to study the cell-type inherent function of autophagy in eosinophils. Surprisingly, and in contrast to other immune cells, eosinophils enhance effector functions when autophagy is impaired. Therefore, drug-induced inhibition of autophagy in chronic inflammatory eosinophilic diseases does not seem to be indicated because of two possible unwanted effects: (1) reduced epithelial barrier function and (2) increased eosinophil-mediated immunopathology. In contrast, however, blocking the autophagic pathway in eosinophils might be beneficial in eosinophilic tumors. Therefore, pharmacological impairment of autophagy might also be an option for the therapy of eosinophilic leukemias. Clearly, additional experimental work, including the analyses of human cells and tissues, is required to identify drug targets suitable for the modulation of the autophagic pathway as preventive or therapeutic intervention in eosinophilic diseases and other human pathologies.
